# Post-Translational Regulation and Trafficking of the Granulin-Containing Protease RD21 of *Arabidopsis thaliana*

**DOI:** 10.1371/journal.pone.0032422

**Published:** 2012-03-02

**Authors:** Christian Gu, Mohammed Shabab, Richard Strasser, Pieter J. Wolters, Takayuki Shindo, Melanie Niemer, Farnusch Kaschani, Lukas Mach, Renier A. L. van der Hoorn

**Affiliations:** 1 The Plant Chemetics Lab, Chemical Genomics Centre of the Max Planck Society, Max Planck Institute for Plant Breeding Research, Cologne, Germany; 2 Department of Applied Genetics and Cell Biology, University of Natural Resources and Life Sciences, Vienna, Austria; University of Hyderabad, India

## Abstract

RD21-like proteases are ubiquitous, plant-specific papain-like proteases typified by carrying a C-terminal granulin domain. RD21-like proteases are involved in immunity and associated with senescence and various types of biotic and abiotic stresses. Here, we interrogated Arabidopsis RD21 regulation and trafficking by site-directed mutagenesis, agroinfiltration, western blotting, protease activity profiling and protein degradation. Using an introduced *N*-glycan sensor, deglycosylation experiments and glyco-engineered *N. benthamiana plants*, we show that RD21 passes through the Golgi where it becomes fucosylated. Our studies demonstrate that RD21 is regulated at three post-translational levels. Prodomain removal is not blocked in the catalytic Cys mutant, indicating that RD21 is activated by a proteolytic cascade. However, RD21 activation in Arabidopsis does not require vacuolar processing enzymes (VPEs) or aleurain-like protease AALP. In contrast, granulin domain removal requires the catalytic Cys and His residues and is therefore autocatalytic. Furthermore, SDS can (re-)activate latent RD21 in Arabidopsis leaf extracts, indicating the existence of a third layer of post-translational regulation, possibly mediated by endogenous inhibitors. RD21 causes a dominant protease activity in Arabidopsis leaf extracts, responsible for SDS-induced proteome degradation.

## Introduction

RD21 (Responsive-to-Desiccation-21, At1g47128) of *Arabidopsis thaliana* belongs to an intriguing class of papain-like Cys proteases (PLCPs), typified by the presence of a C-terminal granulin domain [1], [2]. Recent studies demonstrated that RD21-like proteases are an important component of the plant immune response. Mutant *rd21* lines have enhanced susceptibility for the necrotrophic pathogen *Botrytis cinerea* [3], and silencing of the *RD21* ortholog in *Nicotiana benthamiana* renders the plant susceptible for the oomycete pathogen *Phytophthora infestans* [4], [5].

**Figure 1:**
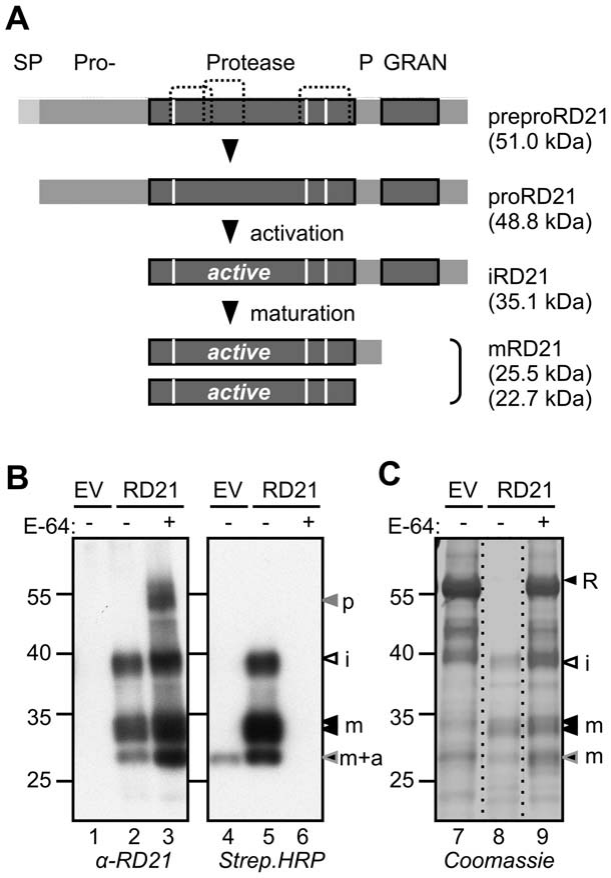
RD21 characteristics and expression by agroinfiltration. **A**, Maturation of RD21. RD21 is encoded as a preproprotease with a signal peptide (SP) for entering the secretion pathway and a prodomain (pro) that keeps the protease inactive until it is removed. RD21 exists in two active isoforms: the intermediate (i) RD21 carries a granulin (GRAN) domain, and the mature (m) RD21 consists of only the protease domain, with or without the proline-rich domain. The theoretical molecular weights are indicated between brackets. White lines, catalytic residues; dashed lines, putative disulphide bridges. **B**, RD21 is active upon agroinfiltration in different isoforms. RD21 was transiently expressed in *Nicotiana benthamiana*. Leaf extracts were generated at three days post-infiltration in the presence or absence of an excess E-64 and labelled with DCG-04 and compared to the empty vector (EV) control. Proteins were analyzed on protein blots with streptavidin-HRP and anti-RD21 antibodies. p, proRD21; a, NbALP; I, intermediate RD21; m, mature RD21. **C**, RD21 degrades rubisco during extraction. *N. benthamiana* were agroinfiltrated with empty vector or RD21-expressing vectors. Extracts were generated in the absence or presence of E-64. Proteins were separated by SDS-PAGE and detected by coomassie staining. R, rubisco (large subunit). The 55 kDa marker protein migrates consistently faster, e.g. when compared to the 50 kDa rubisco protein.

Relatively little is known about the regulation of RD21 activity. Arabidopsis RD21 is composed of five domains: a signal peptide; an autoinhibitory prodomain; the protease domain; a proline-rich domain; and a granulin domain (**Figure 1A**). The granulin domain shares homology to granulins/epithelin in animals, which are growth hormones that are released upon wounding [6]. RD21 matures through two proteolytic steps (**Figure 1A**) [2]. The autoinhibitory prodomain is removed during RD21 activation, resulting in intermediate RD21 (iRD21), which carries the granulin domain. The granulin domain is removed during the subsequent maturation step, resulting in mature RD21 (mRD21).

Arabidopsis RD21 is present in the vacuole and in Endoplasmic Reticulum (ER)-bodies [2], [7], [8]. ER bodies are ER-derived vesicles that occur in cotyledons and in wounded adult leaves, and fuse with the vacuole upon osmotic stress [7]. The presence of RD21 in ER-bodies suggests that RD21 traffics from the ER directly to the vacuole. However, in another study, RD21 was found to traffic through the Golgi to lytic vacuoles [9]. Two endogenous inhibitors have been proposed to regulate RD21 activity. The cytoplasmic Arabidopsis serpin AtSerpin1 irreversibly inhibits RD21 in leaf extracts [10], whereas protein disulphide isomerase-5 (PDI5) binds and inhibits RD21 and accompanies RD21 through the ER and Golgi to the lytic vacuoles [9].

RD21 activity has been detected in Arabidopsis leaf extracts using protease activity profiling [11]. Protease activity profiling is based on the use of a small molecule probe that reacts covalently and irreversibly with the catalytic Cys residue of the protease in a mechanism-dependent manner [12]. DCG-04 is a probe for RD21 and other PLCPs and is a biotinylated derivative of PLCP inhibitor E-64, which carries an epoxide ring that traps the nucleophilic attack by the active site cysteine residue [13]. DCG-04 labeled proteins can be detected on protein blots using streptavidin-HRP (horse radish peroxidase) and purified and identified by mass spectrometry. DCG-04 has been used frequently in plant science, e.g. in studies on serpins [10], senescence [14], and immunity [4], [15]–[20].

Emerging roles of RD21-like proteases in immunity, and their association with various types of stress, prompted us to subject RD21 to further biochemical characterization. In this study, we addressed a series of questions regarding location, activation, maturation and regulation of (mutant) RD21 upon expression in plants. These studies show that RD21 traffics through the Golgi and reveal three consecutive layers of post-translational regulation of RD21. The studies also illustrate the use of agroinfiltration as a protein production platform, and the reliability of DCG-04 profiling as a tool to detect active protease isoforms.

## Results

### Activity and accumulation of agroinfiltrated RD21

We transiently overexpressed RD21 by agroinfiltration of leaves of *N. benthamiana* in the presence of silencing inhibitor p19 to boost the expression levels [21], [22]. Leaf extracts were generated in the presence or absence of PLCP inhibitor E-64, labeled with biotinylated DCG-04 and (labeled) proteins were separated on protein gels and analyzed using anti-RD21 antibody, streptavidin-HRP and coomassie staining.

The anti-RD21 protein blot shows strong signals upon RD21 overexpression, when compared to the empty vector (EV) control (**Figure 1B**, lanes 1 and 2). These signals consist of a 40 kDa iRD21 signal and three signals at 30 kDa, representing different mRD21 isoforms. Adding E-64 during extraction increases the intensities of all these RD21 signals on the protein blot, and reveals an extra 50 kDa signal that probably represents proRD21 (**Figure 1B**, lane 3). Detection with streptavidin-HRP to display biotinylated proteins demonstrates that iRD21 and mRD21 are covalently labeled by DCG-04 (**Figure 1B**, lane 5). No biotinylated signals appear upon preincubation with E-64 (**Figure 1B**, lane 6), showing that the reaction with DCG-04 can be prevented by preincubation with E-64. One biotinylated 25 kDa signal is also present in the empty-vector control (**Figure 1B**, lane 4). This probably represents the endogenous aleurain-like protease from *N. benthamiana* (see below).

Detection of proteins by coomassie staining shows that the 55 kDa rubisco signal disappears upon RD21 expression (**Figure 1C**, lane 8). Adding E-64 during extraction prevents degradation of the rubisco signal (**Figure 1C**, lane 9), demonstrating that this degradation occurs *in vitro*, during protein extraction. Detection of rubisco on coomassie-stained protein gels therefore provides a simple assay for proteolytic activity of RD21. Interestingly, the major proteins that resist this degradation are proteins that coincide in size with iRD21 and mRD21 (**Figure 1C**, lane 8). The absence of these proteins in the empty vector control indicates that these signals indeed represent RD21, demonstrating that iRD21 and mRD21 isoforms accumulate in agroinfiltrated plants in sufficient amounts to be detected by coomassie staining (**Figure 1C**, lanes 7–9). Taken together, these data demonstrate that agroinfiltrated RD21 accumulates as active iRD21 and mRD21 isoforms that degrade rubisco in extracts.

### MS analysis of biotinylated RD21

**Figure 2:**
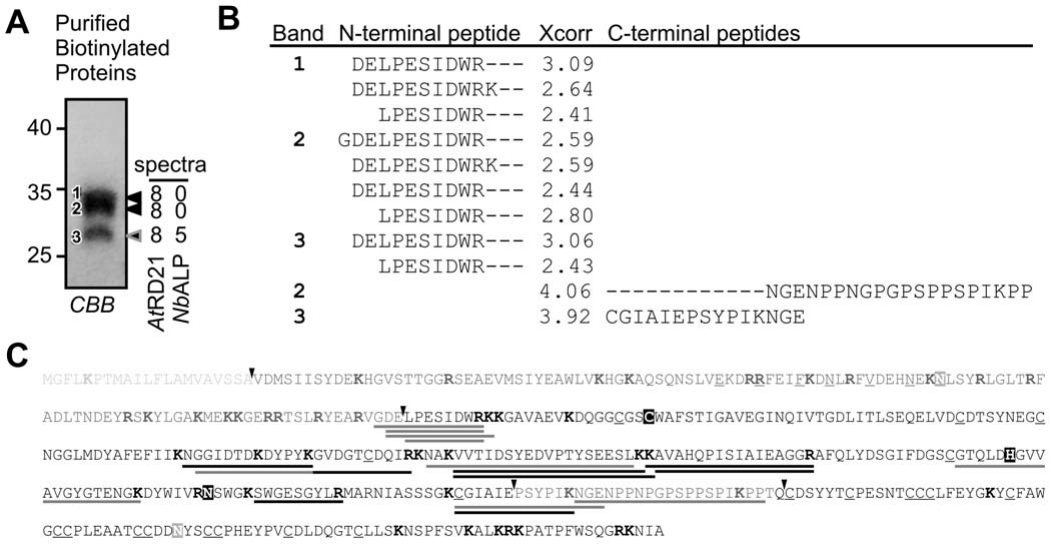
MS-analysis of purified biotinylated proteins. **A**, Extracts from leaves transiently expressing RD21 were labeled with DCG-04. Biotinylated proteins were purified, separated on protein gels and stained by coomassie (CBB). Three bands were excised, treated with trypsin and released peptides were analyzed by LC-MS/MS. iRD21 precipitated during purification and could not be analyzed. Shown are the number of different tryptic peptides with X-correlation values above 2.5. **B**, Analysis of the N- and C-termini. Half-tryptic peptides with X-correlation values above 2.5 were extracted from the LC-MS/MS data obtained as described in (A). Xcorr >2.5, good; >3.5, excellent; >4.5 no doubt. **C**, Identified peptides mapped on the RD21 protein sequence. Indicated are the signal peptide (light grey); prodomain (dark grey) with ERFNIN motif (underlined) and one putative *N*-glycosylation site (boxed grey); protease domain (black) with catalytic residues Cys-His-Asn (boxed) and conserved cysteines (underlined); proline-rich domain (grey); and granulin domain (black) with conserved cysteines (underlined) and one putative *N*-glycosylation site (boxed grey). Tryptic and half tryptic peptides with X-scores >2.5 are underlined with black and grey lines, respectively. Basic residues (trypsin cleavage sites) are printed bold. Triangles, transition between domains.

To confirm the identity of the proteins labeled by DCG-04, biotinylated proteins were purified on streptavidin beads, separated on protein gels and analyzed by mass spectrometry (**Figure 2A**). The iRD21 signal was lost during the purification since iRD21 tends to precipitate [2], [11], [23]. The doublet signal at 30 kDa (bands 1 and 2) yielded multiple tryptic peptides derived from the RD21 protease domain, confirming that these polypeptides represent mRD21 isoforms (**Figure 2A** and **Figure S1**). The 25 kDa signal (band 3) not only contained peptides of the protease domain of RD21, but also peptides from an aleurain-like protease of *N. benthamiana* (NbALP, **Figure S1**). The presence of NbALP in the 25-kDa band indicates that the biotinylated signal in the empty vector control is also caused by NbALP (**Figure 1B**, lane 4). The RD21-derived tryptic peptides cover 40% of the sequence of the RD21 protease domain (**Figure 2C** and **Figure S1**). No tryptic peptides of the signal peptide, prodomain or granulin domain were found, consistent with the absence of these domains in mRD21. Furthermore, the peptide carrying the catalytic Cys was also not detected. This catalytic Cys resides in a 60-residue tryptic peptide (MW 6500 Da), which is too large to be detected, especially when biotinylated.

To determine the N- and C-termini of the different RD21 isoforms, we searched the LC-MS/MS data for half-tryptic peptides. Half tryptic peptides corresponding to N-terminal sequences DELPE and LPE were found in all three samples, and an additional N-terminal sequence GDELPE was found in sample 2 (**Figure 2B**). This indicates that there is a slight variation at the N-terminus of RD21, but this variation does not explain the mass difference between the three signals. Half-tryptic peptides from C-termini were identified corresponding to two positions, but only with one peptide each (**Figure 2B**). The sequences of these peptides suggest that the upper doublet signals at 30 kDa (bands 1 and 2) carry the proline-rich domain, and the lower 25 kDa signal (band 3) lacks most of the proline-rich domain.

### N-glycosylation analysis reveals that RD21 passes through the Golgi apparatus

Since RD21 was originally reported to accumulate in ER bodies that directly bud from the ER and fuse with the vacuole [2], we studied translocation of RD21 upon agroinfiltration by monitoring the *N*-glycosylation status as a sensor for Golgi transit since maturation of *N*-glycosylation in the Golgi leaves unique traces in the *N*-glycans, displayed by differential sensitivity for deglycosylation enzymes like EndoH and PNGaseF [24].

**Figure 3:**
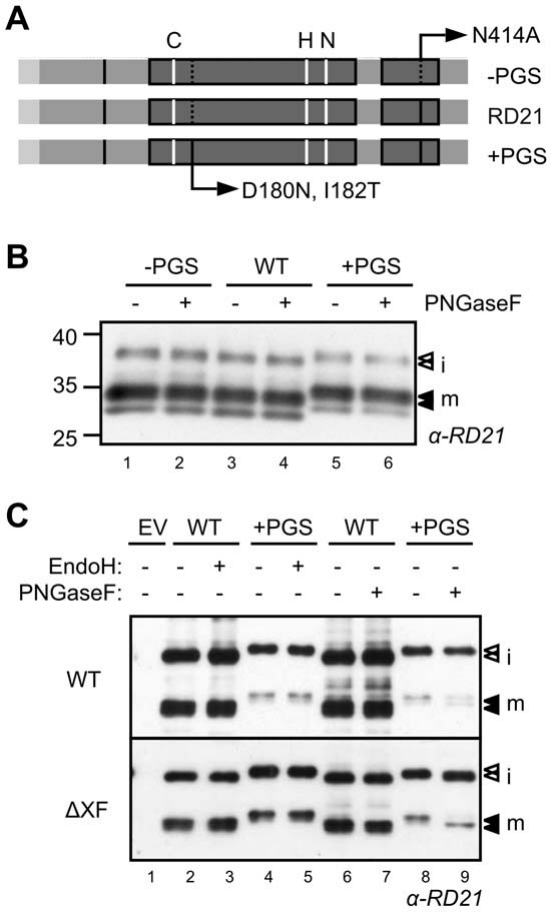
*N*-glycosylation of RD21 demonstrates a Golgi maturation route. **A**, Explanation of the generated *N*-glycosylation mutants. RD21 carries a putative *N*-glycosylation site (PGS) in the prodomain and the granulin domain (black stripes). The second PGS is removed in the –PGS mutant by introducing an N414A substitution. A PGS was introduced in the protease domain of the +PGS mutant by the substitutions D180N and I182T. This PGS is present at the same site in several RD21 orthologs. **B**, The size-shift of the +PGS mutant is not reverted by PNGaseF treatment after expression in wild-type *N. benthamiana* plants. –PGS (pJW03), WT (pRH628) and +PGS (pMS48) were expressed by agroinfiltration and protein extracts were treated with and without PNGaseF. Proteins were separated by SDS-PAGE and analyzed by immunoblotting using RD21 antiserum. **C**, PNGaseF sensitivity is gained by expression of RD21 in glyco-engineered *N. benthamiana* plants. Wild-type RD21 (WT) and the +PGS mutant RD21 (+PGS) were expressed by agroinfiltration into wild-type *N. benthamiana* (WT) and transgenic *N. benthamiana* plants silenced for β-1,2-xylosyltransferase and α-1,3-fucosyltransferase (ΔXF, [27]). Protein extracts were treated with and without EndoH or PNGaseF, separated by SDS-PAGE and analyzed by immunoblotting using RD21 antiserum.

RD21 contains one putative *N*-glycosylation site (PGS, consensus sequence NxS/T) in the prodomain, and one in the granulin domain (**Figures 2C** and **3A**). To test if this second PGS of RD21 is glycosylated, we performed deglycosylation experiments using PNGaseF. However, PNGaseF treatment did not shift the MW of wild-type RD21 (**Figure 3B**, lane 4). Furthermore, we generated the N414A mutant of RD21 which lacks the PGS in the granulin domain (**Figure 3A**). This mutant has a similar MW as WT RD21, and the MW is also unaltered by PNGaseF treatment (**Figure 3B**, lanes 1–2). This indicates that the natural PGS in the granulin domain is not *N*-glycosylated.

To create an *N*-glycan reporter, we introduced a PGS in the protease domain by introducing D180N and I182T substitutions, thereby creating a PGS at amino acid position 180 (+PGS mutant, **Figure 3A**). A PGS at this position is also present in the granulin-containing RD21-like proteins of *Populus trichocarpa* (gi|224103634 and gi|224056176), *Picea stichensis* (gi|116786779) and *Pseudotsuga mendiesii* (gi|1208549). Importantly, western blot analysis show that all isoforms of the +PGS RD21 mutant are slightly larger than the corresponding isoforms of wild-type RD21, consistent with *N*-glycosylation of the protease domain (**Figure 3B**, lane 5). PNGaseF treatment of this mutant isoform, however, does not convert RD21 into a faster migrating protein, indicating that the *N*-glycosylation of the +PGS mutant is PNGaseF-insensitive. PNGaseF can remove the entire *N*-glycan chain from glycosylated Asn residues, but not when the first GlcNAc is alpha-1,3-fucosylated [25]. Fucosylation occurs in the medial Golgi and is mediated by core α-1,3-fucosyltransferase [26]. The PNGaseF-insensitivity of the +PGS mutant RD21 therefore indicates that this mutant RD21 is core alpha-1,3-fucosylated and thus has passed through the medial Golgi.

To test if the +PGS mutant is indeed α-1,3-fucosylated, we took advantage of transgenic *N. benthamiana* plants silenced for β-1,2-xylosyltransferase and α-1,3-fucosyltransferase (ΔXF plants, [26]). Transient expression of the +PGS mutant using agroinfiltration in these ΔXF plants results in protein accumulating with an elevated MW when compared to WT RD21, consistent with *N*-glycosylation (**Figure 3C**, lanes 4 and 8). However, the MW of the isoforms of the +PGS mutant shift downwards upon PNGaseF treatment when expressed in ΔXF plants, but not when expressed in WT plants (**Figure 3C**, lane 9). A small proportion of mRD21 of the +PGS mutant seems PNGaseF sensitive (**Figure 3C**, lanes 8 and 9), perhaps because not all mRD21 is fucosylated. The MW shift does not occur when treated with EndoH, which can only remove *N*-glycans that have not undergone processing in the Golgi (**Figure 3C**, lane 5). Taken together, these data demonstrate that +PGS mutant RD21 carries a fucosylated *N*-glycan, indicating that this protein passes through the Golgi on its route to the vacuole.

### Activity of granulin-domain deletion mutants

**Figure 4:**
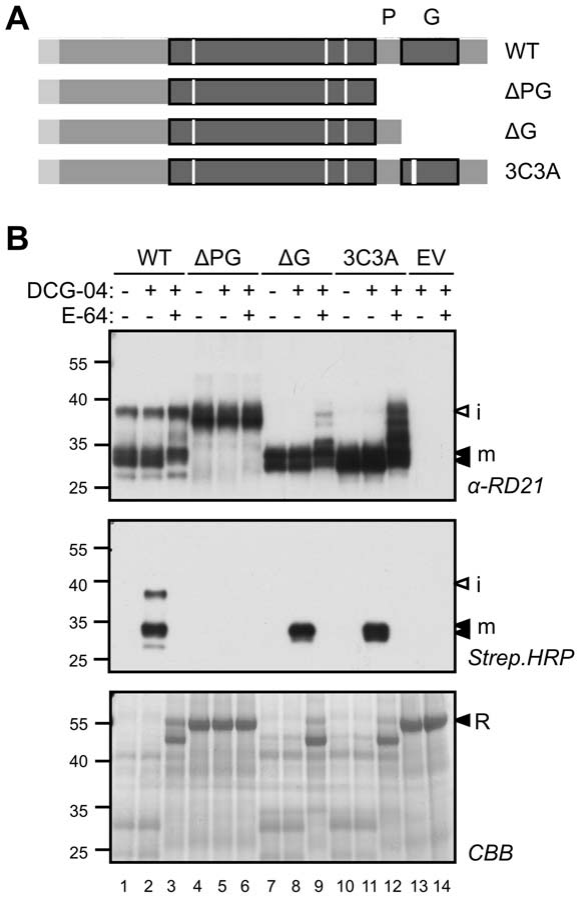
Distinct properties of granulin deletion and destabilization mutants. **A**, Schematic representation of granulin deletion and destabilization mutants. The ΔPG mutant (residues 1–347) lacks both the proline-rich domain (P) and the granulin domain (G), whereas the ΔG mutant (residues 1–374) lacks the granulin domain only. In the 3C3A mutant, a motif of three consecutive Cys residues is replaced by three Ala residues to disrupt three putative disulphide bridges. **B**, Accumulation, labeling and activity of the RD21 deletion and destabilization mutants. The (mutant) RD21 and empty vector (EV) controls were transiently expressed by agroinfiltration in *N. benthamiana* and extracts were labeled with 0.2 µM DCG-04 for 1 hour. RD21 protein levels, labeling, and rubisco (R) degradation were detected using anti-RD21 antibody, streptavidin-HRP and coomassie staining, respectively.

We next generated and tested several C-terminal mutants of RD21 (**Figure 4A**). Interestingly, when both the granulin domain and the proline-rich domain are deleted (ΔPG), a 37 kDa protein accumulates on anti-RD21 blots, but this protein can not be labeled by DCG-04, and does not degrade rubisco, demonstrating that the ΔPG mutant protein is inactive (**Figure 4B**, lanes 4–6). Importantly, the large molecular weight (MW) of this ΔPG protein indicates that it represents the protease domain of RD21 fused to the prodomain (residues 22–347). In other words, deletion of both the granulin and proline-rich domains results in an RD21 precursor that cannot be activated.

To investigate if the granulin domain is required for prodomain removal and RD21 activation, we generated a deletion mutant lacking this domain (ΔG), and a mutant in which three adjacent Cys residues in the granulin domain are replaced by Ala residues (3C3A). The latter mutant is aimed at destabilizing the granulin domain, since these three Cys residues are presumably involved in three disulphide bridges that stabilize the granulin domain [6]. Both the ΔG and 3C3A mutants accumulate as 30 kDa proteins that can be labeled by DCG-04 and degrade rubisco (**Figure 4B**, lanes 7–12). Thus, deletion or destabilization of the granulin domain both result in an active mRD21 protease. These data indicate that the proline-rich domain, or at least part of it, has to be present for prodomain removal.

### Activity, activation and maturation of catalytic RD21 mutants

**Figure 5:**
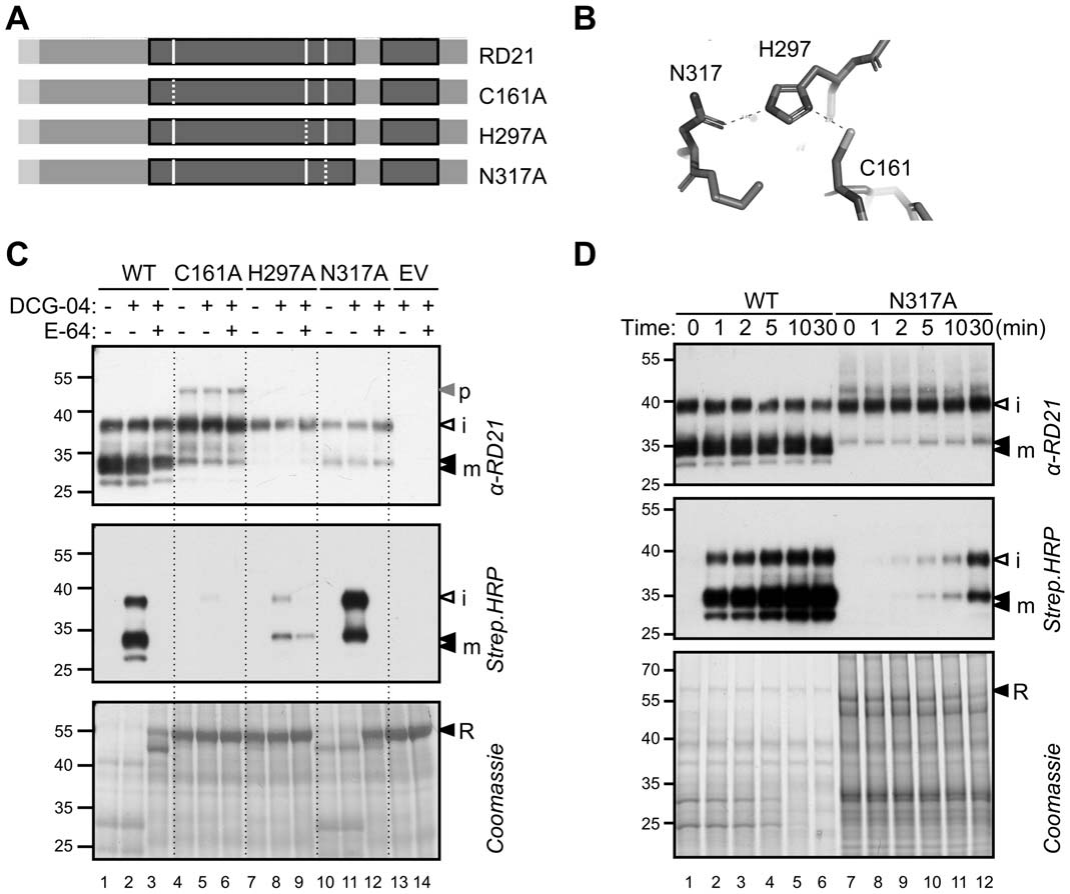
Properties of RD21 catalytic mutants. **A**, Summary of the mutants in the catalytic triad. Each of the three catalytic residues was substituted by Ala to generate three RD21 mutants: C161A, H297A and N317A. **B**, The catalytic triad in RD21. RD21 was modelled on 1S4V using PyMol. The catalytic residues are at 2.6 Å distances from each other (dashed lines). **C**, Accumulation, labeling and activity of the RD21 catalytic mutants. The (mutant) RD21 and empty vector (EV) controls were transiently expressed by agroinfiltration in *N. benthamiana* and extracts were labeled with 0.2 µM DCG-04 for 1 hour. RD21 protein levels, DCG-04 labeling, and rubisco (R) degradation were detected using anti-RD21 antibody, streptavidin-HRP and coomassie staining, respectively. Pro- (p), intermediate (i) and mature (m) isoforms of RD21 were detected. **D**, Time course of labeling of WT and N317A RD21. Extracts from leaves overexpressing WT and N317A RD21 were incubated at pH 6 with 0.2 µM DCG-04 for various incubation times. RD21 protein levels and DCG-04 labeling were detected using anti-RD21 antibody and streptavidin-HRP, respectively. Rubisco degradation was detected by coomassie staining.

The RD21 protease domain contains three important residues in the catalytic site: Cys161, His297 and Asn317 (**Figure 5A**). Together these residues compose the catalytic triad (**Figure 5B**), which deprotonate the catalytic Cys so that it can make a nucleophilic attack on the carbonyl in the peptide substrate. Mutagenesis of the catalytic Cys (C161A) results in an RD21 protein that poorly reacts with DCG-04, and is unable to degrade rubisco (**Figure 5C**, lanes 5–7). Notably, the C161A mutant accumulates mostly as iRD21, indicating that the granulin domain removal requires the catalytic Cys residue. The remaining processing of iRD21 into mRD21 might be caused by endogenous RD21-like proteases of *N. benthamiana*. Furthermore, despite the absence of the catalytic Cys, a weak, E-64-competable biotinylation occurs of iRD21 (**Figure 5C**, lane 5), indicating that some DCG-04 can still react, possibly with the Ser residue that precedes the catalytic Cys.

The H297A mutant is inactive in rubisco degradation, reacts poorly with DCG-04, and accumulates mostly as iRD21 (**Figure 5C**, lanes 7–9). In contrast, the N317A mutant degrades rubisco, and accumulates as iRD21 and mRD21, which are both labeled by DCG-04 (**Figure 5C**, lanes 10–13). The ratio between iRD21 and mRD21 is increased for the N317A mutant when compared to wild-type RD21, indicating that conversion of iRD21 into mRD21 is reduced in this mutant. Taken together, these data indicate that the conversion of iRD21 into mRD21 largely depends on the catalytic activity of RD21 itself.

To investigate the activity of the N317A mutant further, we performed time-courses of labeling and rubisco degradation of WT and N317A RD21. These assays clearly demonstrate that the N317A mutant has a reduced capacity for rubisco degradation and is much slower labeled by DCG-04 (**Figure 5D**). In conclusion, although some of the active-site mutants can be labeled by DCG-04 and degrade rubisco, they have reduced activities in the order: C161A<H297A<N317A<WT.

### PLCP activity in protease-deficient plants

The prodomain removal of the RD21 C161 mutant indicates that other proteases activate RD21. To investigate RD21 activation by candidate proteases in Arabidopsis, we performed protease activity profiling on leaf extracts of various mutant Arabidopsis plants. For this analysis we used the *aalp-1* mutant [28], which lacks AALP, a vacuolar marker protease [29], and *q-vpe*, a quadruple mutant lacking all four vacuolar processing enzymes [30]. We also included the *rd21-1* mutant [28] and the *rd21-1/aalp-1* double mutant, which was generated by crossing.

**Figure 6:**
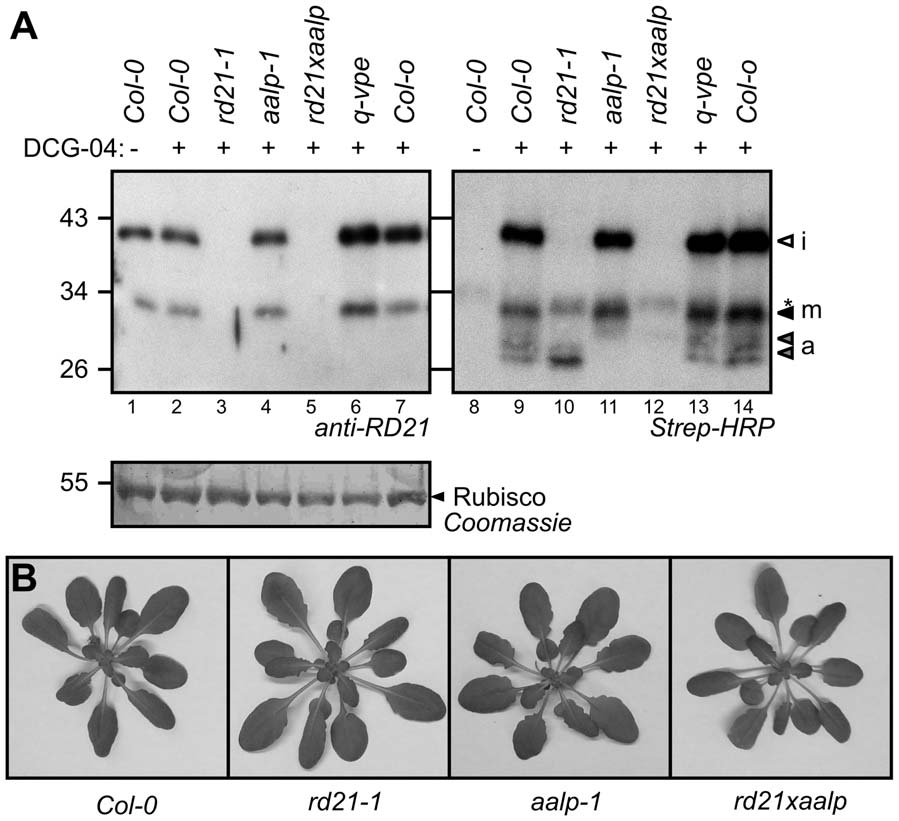
Protease mutants do not display altered protease processing or impaired plant development. **A**, RD21 processing is normal in *vpe* and *aalp* mutants, and AALP processing is normal in *rd21-1* and *vpe* mutants. Leaf extracts of 5-week old (mutant/transgenic) Arabidopsis plants were labeled at pH6. RD21 and biotinylated proteins were detected on protein blots using anti-RD21 antibody or streptavidin-HRP, respectively. Included are: wild-type (*Col-0*); *rd21-1* knockout plants (*rd21-1*), *aalp-1* knockout plants (*aalp-1*), *rd21-1/aalp-1* double knockout plants (*rd21xaalp*); *vpe* quadruple knockout line (*q-vpe*). *, endogenously biotinylated protein. **B**, Despite reduced protease activities, *rd21-1* and *aalp-1* single and double mutants do not have phenotypes under normal growth conditions. Plants were grown for 7 weeks under short-day conditions before pictures were taken.

Western blot detection with anti-RD21 antibody shows signals at 30 and 40 kDa in leaf extracts of ecotype *Colombia-0* (*Col-0*) plants, representing mRD21 and iRD21, respectively (**Figure 6A**, lanes 1 and 2; [2]). These RD21 signals are absent in the *rd21-1* and *rd21-1/aalp-1* mutants (**Figure 6A**, lanes 3 and 5), demonstrating that the RD21 antibody is specific and that the mutant lines are true null mutants. Importantly, the western blot shows normal 30 and 40 kDa signals in both the *aalp-1* and *q-vpe* lines (**Figure 6A**, lanes 4 and 6), demonstrating that VPEs and AALP proteases are not required for RD21 processing.

Protease activity profiling on leaf extracts of *Col-0* plants with DCG-04 causes signals at 25, 30 and 40 kDa, which are absent in the no-probe-control (**Figure 6A**, lanes 8 and 9). The 30 and 40 kDa signals are absent in the *rd21-1* and *rd21-1/aalp-1* mutant lines (**Figure 6A**, lanes 10 and 12), indicating that these signals are caused by RD21. The 25 kDa signal is absent in the *aalp-1* and *rd21-1/aalp-1* mutant lines (**Figure 6A**, lanes 11 and 12), indicating that this signal is caused by AALP. Only weak 30 kDa signals are remaining in the *rd21-1/aalp-1* double mutants, indicating that these mutants have strongly reduced overall PLCP activity. These data also illustrate that no upregulated PLCP activities occur that can function redundantly with RD21 or AALP. Importantly, the 25 kDa signal representing AALP is unaltered in the *rd21-1* and *q-vpe* mutant lines (**Figure 6A**, lanes 10 and 13), demonstrating that these proteases are not required for AALP processing. In conclusion, these experiments show that AALP is dispensable for the activation of RD21 and *vice versa*. Furthermore, our data argue against a role of VPEs in activation of AALP or RD21.

The strongly reduced PLCP activities in the *rd21-1* and *aalp-1* mutants, and especially in the *rd21-1/aalp-1* double mutants, suggests that these plants may have a phenotype. Surprisingly, however, under normal greenhouse conditions, these plants grow indistinguishable from wild-type *Col-0* plants (**Figure 6B**). This indicates that if there is any critical role of RD21 or AALP in plant growth and development, its manifestation requires different conditions.

### SDS activates latent, endogenous RD21 in leaf extracts

**Figure 7:**
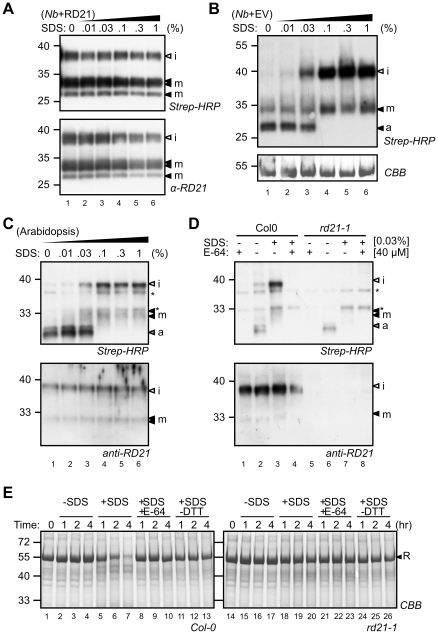
SDS activates endogenous RD21. **A**, The activity of RD21 overexpressed in *N. benthamiana* leaves is not affected by SDS. Leaf extracts were labeled with DCG-04 at various SDS concentrations. RD21 protein levels and biotinylation was detected on protein blots using anti-RD21 antibody and streptavidin-HRP, respectively. **B**, Activation of endogenous PLCPs by SDS treatment of leaf extracts of *N. benthamiana*. **C**, SDS activates RD21 and inactivates AALP (a) in Arabidopsis leaf extracts. Leaf extracts were labeled with DCG-04 in the presence of various SDS concentrations. Biotinylated proteins were detected on protein blots using streptavidin-HRP. **D**, SDS activates latent RD21. Arabidopsis leaf extracts from Col-0 and *rd21-1* plants were labeled with DCG-04 in the presence or absence of 0.1% SDS and 0.1 mM E-64. Biotinylated proteins were detected from protein blots using streptavidin-HRP. *, endogenously biotinylated proteins. **E**, *Ex vivo* degradation of rubisco in Arabidopsis leaf extracts is SDS-dependent and mediated by RD21. Leaf extracts of Arabidopsis *Col-0* and *rd21-1* mutant plants were incubated at room temperature for 0, 1, 2 and 4 hours in the presence and absence of 0.04% SDS, 0.02 mM E-64 or 1 mM DTT. Rubisco (R) was detected by staining protein gels with coomassie (CBB).

An RD21-like protease from maize was previously found to be activated by SDS [31]. Here, we tested if Arabidopsis RD21 can also be activated by SDS. However, when extracts of RD21-expressing *N. benthamiana* leaves were incubated with various SDS concentrations, labeling with DCG-04 occurred irrespective of the SDS concentration (**Figure 7A**). Interestingly, however, we noticed that in the empty-vector control, SDS activates a 35 kDa endogenous protein, presumably the endogenous *N. benthamiana* ortholog of RD21 (**Figure 7B**). These data indicate the overexpressed RD21 is insensitive to SDS-mediated activation, but endogenous RD21 is latent and can be activated by SDS.

To investigate if endogenous RD21 is also latent in Arabidopsis, we labeled Arabidopsis leaf extracts with DCG-04 at various concentrations of SDS. Similar to *N. benthamiana* extracts, this revealed that protease activities change in the presence of increasing concentrations of SDS (**Figure 7C**). The 25 kDa AALP signal disappears at SDS concentrations above 0.03% (**Figure 7C**). In contrast, 30 and 40 kDa signals have the highest intensity at 0.1% SDS (**Figure 7C**). Labeling of the SDS-induced signals by DCG-04 (**Figure 7D**, lane 1) can be competed by adding an excess E-64, indicating that labeling is specific (**Figure 7D**, lane 4). The 40 and 30 kDa signals are absent in SDS-treated extracts of *rd21-1* mutant plants (**Figure 7D**, lane 7), indicating that these signals are derived from RD21, and represent iRD21 and mRD21, respectively.

The increase in DCG-04 labeling upon SDS treatment is correlated with a destabilisation of rubisco in Arabidopsis leaf extracts. When incubated for one or more hours at room temperature in the presence of SDS, rubisco is markedly degraded in leaf extracts of wild-type plants (**Figure 7E**, lanes 5–7). Rubisco degradation is absent when SDS or DTT is omitted during incubation (**Figure 7E**, lanes 2–4 and 11–13), or when E-64 is added (**Figure 7E**, lanes 8–10). Importantly, SDS-induced rubisco degradation does not occur in leaf extracts of *rd21-1* mutant plants (**Figure 6E**, lanes 18–20), demonstrating that RD21 is responsible for SDS-induced rubisco degradation. This indicates that RD21 accounts for most artifactual proteolysis in Arabidopsis leaf extracts during extraction in the presence of SDS and reducing agent.

## Discussion

Emerging roles of RD21-like proteases in immunity, and their association with various types of stress, prompted us to subject RD21 to further biochemical characterization. These studies showed that RD21 traffics through the Golgi and revealed three layers of post-translational regulation: trans-activation, autocatalytic maturation, and latency.

### RD21 exists in different isoforms

We have detected RD21 as proform (proRD21), intermediate isoform (iRD21) and three isoforms of mature RD21 (mRD21). ProRD21 is normally not found in leaf extracts, but this precursor becomes detectable upon extraction in the presence of E-64, suggesting that some proRD21 accumulates in agroinfiltrated leaves and that residual prodomain removal occurs during extraction. However, RD21 in leaf extracts is mostly a mixture of intermediate and mature isoforms. The intermediate iRD21 form aggregates and precipitates during purification [2], [11], [23]. We detected three mRD21 isoforms: a 30 kDa doublet that probably contains the proline-rich domain, and a weaker 25 kDa signal that probably lacks this proline-rich domain. The N-termini of these isoforms are heterogeneous since we detected DELPE and LPE as N-terminal sequences for each isoform and GDELPE for one of them. These N-terminal isoforms may be generated by N-terminal processing by an aminopeptidase after removal of the prodomain. N-terminal processing by dipeptidylaminopeptidase has also been described N-terminus of cathepsin B in animals [32].

Prodomain removal was blocked in the ΔPG deletion mutant, but not in the ΔG deletion mutant. This indicates that the proline-rich domain is required for prodomain removal. However, the presence of a 25 kDa mRD21 isoform suggests that the proline-rich domain is not necessary for protease activity once the prodomain is removed. The granulin domain is not required for prodomain removal and folding since both the ΔG deletion and 3C3A destabilization mutants accumulate as active mRD21.

### N-glycosylation and intracellular transport

Although RD21 contains a putative *N*-glycosylation site (PGS) in the prodomain and another one in the granulin domain, mutagenesis and PNGaseF treatment demonstrated that the PGS in the granulin domain is probably not *N*-glycosylated, possibly because it is buried in the granulin domain and inaccessible for *N*-glycosylation enzymes in the ER [26]. The *N*-glycosylation status of the prodomain has not been addressed in this study.

We introduced a PGS in the protease domain at a position where PGSs occur in other RD21-like proteases. This mutant RD21 becomes indeed *N*-glycosylated, evident from an increased molecular weight of all RD21 isoforms. Deglycosylation experiments with PNGaseF indicated that the *N*-glycan is α-1,3-fucosylated, a hypothesis that was confirmed by transient expression in *N. benthamiana* plants silenced for the respective α-1,3-fucosyltransferase. Fucosylation of glycoproteins is common in plants but does not always occur at all possible sites. *N*-glycosylation of the membrane-resident receptor-like protein Cf-9, for example, occurs at 21 of the 22 PGSs, but all sites are PNGaseF sensitive, indicating that none of these *N*-glycans are fucosylated [33]. Furthermore, Faye et al. [34] reported *N*-glycosylation of two sites on phytohemagglutinin in *Phaseolus vulgaris*: one was fucosylated and the other was not. These data illustrate that fucosylation depends on the accessibility of the *N*-glycan on the folded protein.

RD21 is known to accumulate in the vacuole [2], [8], but the transport route to the vacuole has been controversial. Hayashi et al. [7] detected the protein in ER-bodies that are covered with ribosomes, indicating that RD21 comes directly from the ER. In contrast, Ondzighi et al. [9] showed that RD21 translocates through the Golgi. Our +PGS mutant demonstrates that RD21 passes through the Golgi since the *N*-glycan can not be removed by EndoH, which can only remove oligomannosidic (usually ER-resident) *N*-glycans. Furthermore, the *N*-glycan of RD21 is fucosylated, which requires a fucosyltransferase activity that resides in the medial Golgi [26]. This study demonstrates that the vast majority of RD21 has passed through the Golgi upon agroinfiltration. However, this observation does not mean that RD21 that resides in ER-bodies also traffics through the Golgi. ER-bodies are specific structures that occur in Arabidopsis cotyledons but not in adult leaves, unless these are wounded [7].

### Prodomain removal: in-trans activation?

The majority of the C161A mutant accumulates as iRD21 even though this mutant is inactive in rubisco degradation and DCG-04 labeling. This observation implies that the catalytic Cys is not required for prodomain removal *in planta*. The presence of some proRD21 signal in the C161A mutant may be caused by trapping of proRD21 in the large amount of iRD21 aggregates. A non-autocatalytic prodomain removal is unusual since many PLCPs remove their own prodomain during acidification of their environment, e.g. in the lysosome, the lytic vacuole or the apoplast [35]. However, our findings are consistent with the observation that proRD21 produced in insect cells cannot be activated unless a leaf extract was added [2]. This study, however, did not exclude that a plant-derived non-proteolytic cofactor promotes autocatalytic prodomain removal. The fact that the prodomain is removed from our inactive C161A mutant, indicates that there is another processing protease activating RD21, which implies that RD21 is part of a proteolytic cascade. To our knowledge, proteolytic cascades have not been described in plants before, but are common in animals, e.g. during cancer, where dozens of proteases are trans-proteolytically regulated [36].

Prodomain removal of RD21 is presumably caused by vacuolar proteases. VPE has been proposed as an RD21 activating protease [2], but RD21 matures normally in *vpe* quadruple knockout lines (**Figure 6A**). AALP is another vacuolar protease but RD21 also matures normally in *aalp-1* mutants (**Figure 6A**). These data indicate that the trans activation of RD21 involves proteases that remain to be identified.

In addition to RD21, aleurain-like proteases are also activated by proteolytic processing by a maturating protease [37]. Interestingly, purification of this maturating activity from cauliflower florets resulted in an RD21 ortholog, indicating that RD21 activates an aleurain-like protease [38]. In Arabidopsis, however, RD21 is not required for AALP activation (**Figure 6A**).

### Granulin domain removal: autocatalytic maturation?

The active-site mutants accumulate predominantly as iRD21, indicating that the removal of the granulin domain requires the catalytic activity of RD21. The H297A mutant is not active in rubisco degradation, and is hardly labeled by DCG-04, consistent with the important role of this residue in activating the catalytic Cys residue [39]. Intriguingly, the H297A mutant of RD21 appears rather insensitive to inhibition by E-64. This is in line with the stabilising role of the catalytic His residue in the interaction between E-64 and papain-like cysteine proteinases [40]. In contrast, the N317A mutant is clearly active because it degrades rubisco and is labeled by DCG-04, though its activity is reduced when compared to WT RD21. This indicates that the Asn residue is not an essential catalytic residue. This observation is not unique to RD21, as it was also noted for papain, where a minor effect on proteolytic activity was found upon mutating the catalytic Asn [41].

The removal of the granulin domain is probably caused by cleavage between the proline-rich domain and the granulin domain, since a peptide with the C-terminal sequence -IKPP was found for the most abundant mRD21 isoform. The removal of the proline-rich domain, resulting in the 25 kDa mRD21 isoform, may occur subsequently by further trimming of 30 kDa mRD21, or directly by cleavage of 40 kDa iRD21.

The fate of the granulin domain is unknown. The fact that the granulin domain endures in lytic vesicles when fused to the protease domain, suggests that this is a stable domain that may persist in plants after being cleaved from the protease domain. The importance of the Cys residues in the stabilization of the granulin domain was demonstrated by the 3C3A mutants. In this mutant, three disulphide bridges involving three adjacent Cys residues are disrupted by mutagenesis into three adjacent Ala residues. The 3C3A mutant protein accumulates only in the mRD21 isoform, indicating that the granulin domain is not stable when some of the disulphide bridges are absent.

### SDS activates latent RD21

SDS activates endogenous Arabidopsis RD21 and a presumed RD21 ortholog of *N. benthamiana*, but not agroinfiltrated RD21. The SDS treatment does not change the ratio of iRD21 and mRD21, indicating that both isoforms are activated by SDS and that the granulin domain does not affect latency. We hypothesize that RD21 latency can be explained by the presence of a limited amount of SDS-sensitive endogenous inhibitor.

There are two candidates for these SDS-sensitive non-covalent endogenous inhibitors: PDI5 and cystatins. Protein disulphide isomerase-5 (PDI5) interacts and colocalizes with RD21 [9]. A limited amount of PDI5-like proteins could explain why latency is not detected when the protease is overexpressed. Cystatins have been studied in relation to SDS treatment before. The SDS-activatable RD21 ortholog of maize (CPPIC) has been purified in complex with an endogenous cystatin, which prompted the hypothesis that SDS acts by denaturing the endogenous inhibitor [42]. However, SDS was still required for activation of CPPIC even after removal of the cystatin from the protease complex [43]. This indicates that the interaction with the cystatin causes conformational changes that keep CPPIC latent after cystatin removal, and SDS reverts these conformational changes to activate CPPIC [43]. A cystatin complex with the RD21 ortholog has also been purified from potato tubers [44] and senescing spinach leaves [45]. In case of the spinach RD21 ortholog, the removal of the cystatin by acid treatment was sufficient for activation. Thus, endogenous cystatins are common interaction partners of RD21-like proteases and likely candidates to explain RD21 latency in plant extracts. The limited amount of endogenous cystatins could explain why latency is not detected when the protease is overexpressed.

### Post-translational regulation of RD21 at three levels

In conclusion, we have studied three levels of post-translational regulation of RD21 activity. The first level is the removal of the prodomain. Prodomain removal is essential for activation of RD21 and is not autocatalytic and presumably involves another protease. The second level is the conversion of iRD21 into mRD21 by removal of the granulin domain. This maturation is probably autocatalytic since it requires the catalytic Cys and His residues. This maturation is thought to increase the proteolytic activity since iRD21 resides in aggregates and will have less access to substrates, whereas mRD21 is a soluble protease [2]. The third level of RD21 regulation is represented by the latency of endogenous RD21. This latency is released by adding SDS, and might be caused by the presence of endogenous inhibitors.

### Practical implications of SDS-activation, DCG-04 profiling, and agroinfiltration

The activation of latent RD21 by SDS may also have important practical implications. SDS and DTT are standard components of SDS-PAGE loading buffers and often thought to denature and inactivate proteolytic activities. Our experiments illustrate that these conditions rather activate RD21, causing slow, but significant proteome degradation. Both SDS and DTT are required for this process and proteomes are more stable in extracts from *rd21-1* knockout lines, illustrating that RD21 is a major proteolytic activity affecting proteome stability in leaf extracts.

Our studies show a consistent correlation between DCG-04 labeling and protein degradation. For example, the catalytic C161A and H297A mutants, and the ΔPG deletion mutants are unable to degrade rubisco and are poorly labelled with DCG-04. The catalytic N317A mutant is slow in rubisco degradation and DCG04 labeling when compared to wild-type RD21. Furthermore, SDS-induced rubisco degradation is associated with increased DCG-04 labeling in Arabidopsis leaf extracts. These observations support the notion that protease activity profiling is a reliable prediction of protease activity.

Besides structure-function analysis of RD21, our studies also provide useful observations for agroinfiltration as protein production platform. Production of heterologous proteins in *N. benthamiana* by agroinfiltration for medical purposes has become commercially attractive [46]. Our work shows that even proteolytically active proteins can be overexpressed in *N. benthamiana* to very high levels. The proteins pass through the Golgi, receive the necessary post-translational modifications and accumulate presumably in lytic vacuoles to relatively high concentrations that nevertheless remain harmless for the plant.

## Materials and Methods

### Molecular cloning

A summary of the cloning is provided in **Tables S1** and **S2**. The following plasmids have been described previously: pRH80 [47]; pRH385 [33]; pTP5 [18]. The full length RD21 and the C-terminal deletion mutants were generated by PCR using the cDNA clone U11707 (ABRC) as a template. The introduction of an XhoI site at the end of the open reading frame translates into two (pRH628) and three (pRH629) additional Ser residues at the C-terminus. PCR fragments were subcloned into pRH80 (-derivatives) and the sequence was verified by sequencing. Site-directed mutants were generated by Quick Change (Stratagene) using pRH626 as template and primers summarized in **Table S2**. The verified mutant expression cassettes were shuttled into binary vectors as summarized in **Table S1**, resulting in a collection of binary plasmids (**Table S3**) that all carry a T-DNA with a 35S-driven (mutant) RD21 open reading frame, followed by a terminator of the potato PI-II gene.

### Agroinfiltration

The binary vectors were transformed into *Agrobacterium tumefaciens* GV3501 pMP90 [48] by electroporation and selected on rifampicin (50 µg/ml) and kanamycin (50 µg/ml). The strains were grown in 10 mL LB medium with the antibiotics (rifampicin 50 µg/ml and kanamycin 50 µg/ml) overnight at 28°C. The bacteria were gently centrifuged (3500 rpm for 15 min at room temperature) and resuspended in 10 mM MgCl_2_, containing 0.2 µM acetosyringone. Bacteria were diluted to OD_600_ = 2 and mixed with an equal volume of Agrobacterium carrying a binary plasmid that encodes silencing inhibitor p19 [21] at OD_600_ = 0.4. The mixed cultures were kept at room temperature for 1 hr and infiltrated into the two youngest, fully unfolded leaves of 4-week old *N. benthamiana* plants.

### Extraction and labeling

Leaf discs without main veins of agroinfiltrated *N. benthamiana* leaves at the third day after agroinfiltration were cut into small pieces with a razor blade and ground in 10 mM Tris pH8, containing 5 mM DTT, in an ice-cold mortar. After removing insoluble materials by repeated centrifugation at 20,000 g for 5 min at 4°C, the protein extracts were adjusted to a concentration of 1 mg/mL. 100 µg of protein extracts was labeled in 500 µL 100 mM sodium acetate pH 6, containing 1 mM DTT, with 0.2 µM DCG-04 for 1 hr at room temperature. In no-probe-controls (NPC) 1 µL DMSO was added instead of 1 µL 1 mM DCG-04 in DMSO. In E-64 treatments, an extra 20 µM E-64 was added before adding the probe. The labeling reactions were stopped by adding 1 mL of 100% acetone at −20°C. The proteins were pelleted by centrifugation at 20,000 g for 5 min at 4°C and the protein pellets were dissolved in 50 µL SDS-PAGE loading buffer containing β-mercaptoethanol. The proteins were separated on 12% SDS PAGE gels (10 µg protein per lane) and the detection of biotinylated proteins was done as previously described using streptavidin-HRP (Sigma-Aldrich) [11]. Purification and identification of DCG-04 labeled proteins was performed as described previously [11].

### Deglycosylation with PNGaseF and EndoH

Agrobacteria carrying pRH628, pMS48, or pJW03 were infiltrated into leaves of wild-type *N. benthamiana* (NbWT) and transgenic *N. benthamiana* silenced for both β-1,2-xylosyltransferase and α-1,3-fucosyltransferase (NbΔXF, [27]). Leaf material from NbWT and NbΔXF was harvested 48 h after infiltration, ground in liquid nitrogen and resuspended in 1× phosphate-buffered saline (PBS) plus 1% (v/v) protease inhibitor cocktail. Samples were incubated for 10 min on ice and then centrifuged for 10 min at 10.000 rpm. The supernatant was again centrifuged for 5 min at 10.000 rpm. For PNGaseF digestion, the extract (10 µl) was mixed with 1.5 µL 10× G7 reaction buffer (NEB), 1.5 µL 10% NP-40, and 0.5 units PNGaseF (P0704, NEB) in a total volume of 15 µl. For EndoH digestion, the extract was mixed with 1.5 µL 10× G5 reaction buffer (NEB), and 0.5 units EndoH (P0702, NEB) in a total volume of 15 µl. Controls were treated as described above but the respective enzymes were replaced by water. After incubation of the reaction mixture for 60 minutes at 37°C, SDS-PAGE loading buffer was added and heated to 95°C for 5 minutes. Proteins were then subjected to SDS-PAGE (12%) followed by protein blot analysis with anti-RD21 (1∶5000) antibody.

## Supporting Information

Figure S1 **Identification of DCG-04 labeled proteases from agroinfiltrated** ***N. benthamiana*** **.** DCG-04 labeled proteins were purified, digested with trypsin, and analyzed by mass spectrometry. **A**) Sequences of Arabidopsis RD21 and *N. benthamiana* ALP (Hao et al., 2006). The signal peptide and prodomain are indicated in light and dark grey, respectively. The identified tryptic peptides and catalytic residues are underlined and boldface, respectively. **B**) Summary of the identified tryptic peptides, with individual scores. *, oxidized methionine; **, X correlation value: >1.5 maybe; >2.5 good; >3.5 excellent; >4.5 no doubt. Only tryptic peptides are shown.
(PDF)

Table S1 **Cloning details.**
(PDF)

Table S2 **Primer sequences.**
(PDF)

Table S3 **List of binary plasmids used in this work.**
(PDF)
