# Supplementary material for: Post-Translational Regulation and Trafficking of the Granulin-Containing Protease RD21 of Arabidopsis thaliana
Source: PLoS One. 2012 Mar 2;7(3):e32422. doi: 10.1371/journal.pone.0032422 (PMC3292552; doi:10.1371/journal.pone.0032422)
Supplement: Figure S1 — Identification of DCG-04 labeled proteases from agroinfiltrated N. benthamiana . DCG-04 labeled proteins were purified, digested with trypsin, and analyzed by mass spectrometry. A) Sequences of Arabidopsis RD21 and N. benthamiana ALP (Hao et al., 2006). The signal peptide and prodomain are indicated in light and dark grey, respectively. The identified tryptic peptides and catalytic residues are underlined and boldface, respectively. B) Summary of the identified tryptic peptides, with individual scores. *, oxidized methionine; **, X correlation value: >1.5 maybe; >2.5 good; >3.5 excellent; >4.5 no doubt. Only tryptic peptides are shown. (PDF) [file pone.0032422.s001.pdf]

A

>AtRD21 NP_564497 [*Arabidopsis thaliana*]
MGFLKPTMAILFLAMVAVSSAVDMSIISYDEKHGVSTTGGRSEAEVMSIYEAWLVKHGKAQSQNSLVEK
DRRFEIFKDNLRFVDEHNEKNLSYRLGLTRFADLTNDEYRSKYLGAKMEKKGERRTSLRYEARVGDELP
ESIDWRKKGAVAEVKDQGGCGS**C**WAFSTIGAVEGINQIVTGDLITLSEQELVDCDTSYNEGCNGGLMDY
AFEFIIKNGGIDTDKDYPYKGVDGTCDQIRKNAKVVTIDSYEDVPTYSEESLKKAVAHQPISIAIEAGGRAF
QLYDSGIFDGSCGTQLD**H**GVVAVGYGTENGKDYWIVR**N**SWGKSWGESGYLRMARNIASSSGKCGIAIEP
SYPIKNGENPPNPGPSPPSPIKPPTQCDSYYTCPESNTCCCLFEYGKYCFAWGCCPLEAATCCDDNYSCCP
HEYPVCDLDQGTCLLSKNSPFSVKALKRKPATPFWSQGRKNIA

>NbALP DQ094022 [*Nicotiana benthamiana*]
MSRFSLLLALVVAGGLFAAALAGPATFADENPIRQIVSDGLHELENGILQVVGKTRHALLFARFAHRYGK
RYETVEEIKQRFEVFLDNLKMIRSHNKKGLSYKLGVNEFTDITWDEFRRDRLGAAQNCSATTKGNLKLTN
VVLPETKDWREAGIVSPVKNQGKCGSCWTFSTTGALEAAYGQAFGKGISLSEQQLVDCAGAFNNFGCNG
GLPSQAFEYIKSNGGLDTEEAYPYTGKMGLCKFSSENVGVKVIDSVNITLGAEDELKYAVALVRPVSIAFE
VIKGFKQYKSGVYTSTECGNTPMDVNHAVLAVGYGVENGVPYWLIKNSWGADWGDNGYFKMEMGKN
MCGIATCASYPVVA

B

Band	Peptide sequence	Origin	Xcorr**	dCn
3	K.AVAHQPISIAIEAGGR.A	AtRD21	5.15	0.32
	K.CGIAIEPSYPIK.N	AtRD21	2.52	0.20
	K.DYWIVR.N	AtRD21	2.07	0.01
	K.KAVAHQPISIAIEAGGR.A	AtRD21	5.48	0.45
	K.NGGIDTDKDYPYK.G	AtRD21	4.17	0.37
	K.SWGESGYLR.M	AtRD21	2.69	0.20
	K.VVTIDSYEDVPTYSEESLK.K	AtRD21	5.10	0.50
	K.VVTIDSYEDVPTYSEESLKK.A	AtRD21	4.08	0.27
4	K.AVAHQPISIAIEAGGR.A	AtRD21	4.55	0.39
	K.CGIAIEPSYPIK.N	AtRD21	3.15	0.15
	K.GVDGTCDQIR.K	AtRD21	2.65	0.13
	K.KAVAHQPISIAIEAGGR.A	AtRD21	5.60	0.43
	K.NGGIDTDKDYPYK.G	AtRD21	4.08	0.15
	K.SWGESGYLR.M	AtRD21	2.85	0.09
	K.VVTIDSYEDVPTYSEESLK.K	AtRD21	5.52	0.50
	K.VVTIDSYEDVPTYSEESLKK.A	AtRD21	4.86	0.36
5	K.AVAHQPISIAIEAGGR.A	AtRD21	4.30	0.48
	K.CGIAIEPSYPIK.N	AtRD21	4.28	0.24
	K.DYWIVR.N	AtRD21	2.21	0.07
	K.KAVAHQPISIAIEAGGR.A	AtRD21	5.66	0.51
	K.NGGIDTDKDYPYK.G	AtRD21	3.92	0.38
	K.SWGESGYLR.M	AtRD21	2.83	0.26
	K.VVTIDSYEDVPTYSEESLK.K	AtRD21	5.37	0.46
	K.VVTIDSYEDVPTYSEESLKK.A	AtRD21	4.18	0.35
	K.NM*CGIATCASYPVVA	NbALP	3.51	0,42
	K.NMCGIATCASYPVVA	NbALP	3.03	0,40
	K.SNGGLDTEEAYPYTGK.N	NbALP	4.90	0,47
	K.YAVALVR.P	NbALP	2.73	0,16
	K.YAVALVRPVSIAFEVIK.G	NbALP	5.09	0,36

Figure S1 Identification of DCG-04 labeled proteases from agroinfiltrated *N. benthamiana*. DCG-04 labeled proteins were purified, digested with trypsin, and analyzed by mass spectrometry. **A)** Sequences of Arabidopsis RD21 and *N. benthamiana* ALP (Hao et al., 2006). The signal peptide and prodomain are indicated in light and dark grey, respectively. The identified tryptic peptides and catalytic residues are underlined and boldface, respectively. **B)** Summary of the identified tryptic peptides, with individual scores. *, oxidized methionine; **, X correlation value: >1.5 maybe; >2.5 good; >3.5 excellent; >4.5 no doubt. Only tryptic peptides are shown.
