# Supplementary material for: Post-Translational Regulation and Trafficking of the Granulin-Containing Protease RD21 of Arabidopsis thaliana
Source: PLoS One. 2012 Mar 2;7(3):e32422. doi: 10.1371/journal.pone.0032422 (PMC3292552; doi:10.1371/journal.pone.0032422)
Supplement: Table S1 — Cloning details. (PDF) [file pone.0032422.s002.pdf]

Table S1: Cloning details

Construct	Forward primer	Reverse primer	Restriction enzymes	Acceptor	Plasmid	Restriction enzymes	Acceptor	Binary plasmid
RD21 WT	RD21AF	F05j	NcoI+PstI	pRH80	pRH626	EcoRI + XbaI	pRH385	pRH628
ΔPG	RD21AF	F05i	NcoI+PstI	pRH80	pRH625	EcoRI + XbaI	pRH385	pRH629
ΔG	RD21AF	R135	NcoI+PstI	pRH626	pMS5	EcoRI + XbaI	pTP5	pMS17
3C3A	R136	R137	(Quick Change pRH626)		pMS10	EcoRI + XbaI	pTP5	pMS23
C161A	R121	R122	(Quick Change pRH626)		pMS02	EcoRI + XbaI	pTP5	pMS14
H297A	R127	R128	(Quick Change pRH626)		pMS42	EcoRI + XbaI	pTP5	pMS49
N317A	R129	R130	(Quick Change pRH626)		pMS3	EcoRI + XbaI	pTP5	pMS15
N414A	R133	R134	(Quick Change pRH626)		pMS9	EcoRI + XbaI	pTP5	pJW03
D180N, I182T	R123	R124	(Quick Change pRH626)		pMS4	EcoRI + XbaI	pTP5	pMS48
