# Supplementary material for: Post-Translational Regulation and Trafficking of the Granulin-Containing Protease RD21 of Arabidopsis thaliana
Source: PLoS One. 2012 Mar 2;7(3):e32422. doi: 10.1371/journal.pone.0032422 (PMC3292552; doi:10.1371/journal.pone.0032422)
Supplement: Table S2 — Primer sequences. (PDF) [file pone.0032422.s003.pdf]

Table S2: Primer sequences

**Primer**	**Sequence (5' to 3')**
RD21AF	ATGCCCATGGGGTTCCTTAAGCCAACGATGGCG
F05j	CCCCCTGCAGTTAGCTCGAGGCAATGTTCTTTCTGCCTTG
F05i	CCCCCTGCAGTTAGCTCGAGCTTTCAATCGCGATTCCACATTTTCG
R121	CAGGGTGGTTGCGGGAGTgcTTGGGCGTTTTCAACCATTGG
R122	CCAATGGTTGAAAACGCCCAAgcACTCCCGCAACCACCCTG
R123	CCAGATCGTAACCGGAaACCTAAcAACCTTGTCTGAACAAG
R124	CTTGTTCAGACAAGGTTgTTAGGTtTCCGGTTACGATCTGG
R127	GGAAGTTGTGGAACACAACTAGACgcCGGAGTTGTGGCGGTTGG
R128	CCAACCGCCACAACTCCGgcGTCTAGTTGTGTTCCACAACTTCC
R129	GATTACTGGATTGTGAGAgcCTCATGGGGTAAAAGCTGGGGAG
R130	CTCCCCAGCTTTTACCCCATGAGgcTCTCACAATCCAGTAATC
R133	GCAGCCACTTGCTGTGATGACgcCTATAGTTGCTGCCCTCACGAG
R134	CTCGTGAGGGCAGCAACTATAGgcGTCATCACAGCAAGTGGCTGC
R135	CCCCCTGCAGCTATTGGGTTGGAGGCTTGATGGGAGATGG
R136	CACTTGTCCTGAGAGCAACACTgcTgcTgcTCTGTTTGAGTATGGCAAG
R137	CTTGCCATACTCAAACAGAgcAgcAgcAGTGTTGCTCTCAGGACAAGTG
