# Supplementary material for: Post-Translational Regulation and Trafficking of the Granulin-Containing Protease RD21 of Arabidopsis thaliana
Source: PLoS One. 2012 Mar 2;7(3):e32422. doi: 10.1371/journal.pone.0032422 (PMC3292552; doi:10.1371/journal.pone.0032422)
Supplement: Table S3 — List of binary plasmids used in this work. (PDF) [file pone.0032422.s004.pdf]

Table S3: List of binary plasmids used in this work

**Plasmid**	**Protein**	**Ref**	**Reference**
pTP5	EV	Empty vector	Shabab et al., 2008
pRH628	WT	Wild-type RD21 protein	This work
pRH629	ΔPG	Deletion of pro-rich and granulin domains	This work
pMS17	ΔG	Deletion of granulin domain	This work
pMS23	3C3A	Granulin destabilization mutant	This work
pMS14	C161A	Catalytic Cys mutant	This work
pMS49	H297A	Catalytic His mutant	This work
pMS15	N317A	Catalytic Asn mutant	This work
pJW03	N414A	Putative *N*-glycosylation site removed	This work
pMS48	D180N/I182T	Extra *N*-glycosylation site introduced	This work
